# Supplementary material for: OpenMM 7: Rapid development of high performance algorithms for molecular dynamics
Source: PLoS Comput Biol. 2017 Jul 26;13(7):e1005659. doi: 10.1371/journal.pcbi.1005659 (PMC5549999; doi:10.1371/journal.pcbi.1005659)
Supplement: S1 Source Code — (ZIP) [file pcbi.1005659.s002.zip › OpenMM-7.0.1-Source/docs/api-python/generated/simtk.openmm.openmm.System.html]

System — OpenMM 7.0.1.dev-5e86c4f documentation


# System¶

*class* `simtk.openmm.openmm.``System`(*\*args*)¶
:   This class represents a molecular system. The definition of a System involves four elements:

    The particles and constraints are defined directly by the System object, while forces are defined by objects that extend the Force class. After creating a System, call addParticle() once for each particle, addConstraint() for each constraint, and addForce() for each Force.

    In addition, particles may be designated as “virtual sites”. These are particles whose positions are computed automatically based on the positions of other particles. To define a virtual site, call setVirtualSite(), passing in a VirtualSite object that defines the rules for computing its position.

    `__init__`(*self*) → System¶
    :   \_\_init\_\_(self, other) -> System

        Create a new System.

    Methods

    |  |  |
    | --- | --- |
    | `__init__`((self) -> System) | \_\_init\_\_(self, other) -> System |
    | `addConstraint`((self, particle1, particle2, ...) | Add a constraint to the System. |
    | `addForce`((self, force) -> int) | Add a Force to the System. |
    | `addParticle`((self, mass) -> int) | Add a particle to the System. |
    | `getConstraintParameters`(self, index) | Get the parameters defining a distance constraint. |
    | `getDefaultPeriodicBoxVectors`(self) | Get the default values of the vectors defining the axes of the periodic box (measured in nm). |
    | `getForce`((self, index) -> Force) | getForce(self, index) -> Force |
    | `getForces`() | Get the list of Forces in this System |
    | `getNumConstraints`((self) -> int) | Get the number of distance constraints in this System. |
    | `getNumForces`((self) -> int) | Get the number of Force objects that have been added to the System. |
    | `getNumParticles`((self) -> int) | Get the number of particles in this System. |
    | `getParticleMass`((self, index) -> double) | Get the mass (in atomic mass units) of a particle. |
    | `getVirtualSite`((self, index) -> VirtualSite) | Get VirtualSite object for a particle. |
    | `isVirtualSite`((self, index) -> bool) | Get whether a particle is a VirtualSite. |
    | `removeConstraint`(self, index) | Remove a constraint from the System. |
    | `removeForce`(self, index) | Remove a Force from the System. |
    | `setConstraintParameters`(self, index, ...) | Set the parameters defining a distance constraint. |
    | `setDefaultPeriodicBoxVectors`(self, a, b, c) | Set the default values of the vectors defining the axes of the periodic box (measured in nm). |
    | `setParticleMass`(self, index, mass) | Set the mass (in atomic mass units) of a particle. |
    | `setVirtualSite`(self, index, virtualSite) | Set a particle to be a virtual site. |
    | `usesPeriodicBoundaryConditions`((self) -> bool) | Returns whether or not any forces in this System use periodic boundaries. |

    `getNumParticles`(*self*) → int¶
    :   Get the number of particles in this System.

    `addParticle`(*self*, *mass*) → int¶
    :   Add a particle to the System. If the mass is 0, Integrators will ignore the particle and not modify its position or velocity. This is most often used for virtual sites, but can also be used as a way to prevent a particle from moving.

        |  |  |
        | --- | --- |
        | Parameters: | **mass** (*double*) – the mass of the particle (in atomic mass units) |
        | Returns: | the index of the particle that was added |
        | Return type: | int |

    `getParticleMass`(*self*, *index*) → double¶
    :   Get the mass (in atomic mass units) of a particle. If the mass is 0, Integrators will ignore the particle and not modify its position or velocity. This is most often used for virtual sites, but can also be used as a way to prevent a particle from moving.

        |  |  |
        | --- | --- |
        | Parameters: | **index** (*int*) – the index of the particle for which to get the mass |

    `setParticleMass`(*self*, *index*, *mass*)¶
    :   Set the mass (in atomic mass units) of a particle. If the mass is 0, Integrators will ignore the particle and not modify its position or velocity. This is most often used for virtual sites, but can also be used as a way to prevent a particle from moving.

        |  |  |
        | --- | --- |
        | Parameters: | - **index** (*int*) – the index of the particle for which to set the mass - **mass** (*double*) – the mass of the particle |

    `setVirtualSite`(*self*, *index*, *virtualSite*)¶
    :   Set a particle to be a virtual site. The VirtualSite object should have been created on the heap with the “new” operator. The System takes over ownership of it, and deletes it when the System itself is deleted.

        |  |  |
        | --- | --- |
        | Parameters: | - **index** (*int*) – the index of the particle that should be treated as a virtual site - **virtualSite** (*VirtualSite \**) – a pointer to the VirtualSite object describing it |

    `isVirtualSite`(*self*, *index*) → bool¶
    :   Get whether a particle is a VirtualSite.

        |  |  |
        | --- | --- |
        | Parameters: | **index** (*int*) – the index of the particle to check |

    `getVirtualSite`(*self*, *index*) → VirtualSite¶
    :   Get VirtualSite object for a particle. If the particle is not a virtual site, this throws an exception.

        |  |  |
        | --- | --- |
        | Parameters: | **index** (*int*) – the index of the particle to get |

    `getNumConstraints`(*self*) → int¶
    :   Get the number of distance constraints in this System.

    `addConstraint`(*self*, *particle1*, *particle2*, *distance*) → int¶
    :   Add a constraint to the System. Particles whose mass is 0 cannot participate in constraints.

        |  |  |
        | --- | --- |
        | Parameters: | - **particle1** (*int*) – the index of the first particle involved in the constraint - **particle2** (*int*) – the index of the second particle involved in the constraint - **distance** (*double*) – the required distance between the two particles, measured in nm |
        | Returns: | the index of the constraint that was added |
        | Return type: | int |

    `getConstraintParameters`(*self*, *index*)¶
    :   Get the parameters defining a distance constraint.

        |  |  |
        | --- | --- |
        | Parameters: | **index** (*int*) – the index of the constraint for which to get parameters |
        | Returns: | - **particle1** (*int*) –   the index of the first particle involved in the constraint - **particle2** (*int*) –   the index of the second particle involved in the constraint - **distance** (*double*) –   the required distance between the two particles, measured in nm |

    `setConstraintParameters`(*self*, *index*, *particle1*, *particle2*, *distance*)¶
    :   Set the parameters defining a distance constraint. Particles whose mass is 0 cannot participate in constraints.

        |  |  |
        | --- | --- |
        | Parameters: | - **index** (*int*) – the index of the constraint for which to set parameters - **particle1** (*int*) – the index of the first particle involved in the constraint - **particle2** (*int*) – the index of the second particle involved in the constraint - **distance** (*double*) – the required distance between the two particles, measured in nm |

    `removeConstraint`(*self*, *index*)¶
    :   Remove a constraint from the System.

        |  |  |
        | --- | --- |
        | Parameters: | **index** (*int*) – the index of the constraint to remove |

    `addForce`(*self*, *force*) → int¶
    :   Add a Force to the System. The Force should have been created on the heap with the “new” operator. The System takes over ownership of it, and deletes the Force when the System itself is deleted.

        |  |  |
        | --- | --- |
        | Parameters: | **force** (*Force \**) – a pointer to the Force object to be added |
        | Returns: | the index within the System of the Force that was added |
        | Return type: | int |

    `getNumForces`(*self*) → int¶
    :   Get the number of Force objects that have been added to the System.

    `getForce`(*self*, *index*) → Force¶
    :   getForce(self, index) -> Force

        Get a writable reference to one of the Forces in this System.

        |  |  |
        | --- | --- |
        | Parameters: | **index** (*int*) – the index of the Force to get |

    `removeForce`(*self*, *index*)¶
    :   Remove a Force from the System. The memory associated with the removed Force object is deleted.

        |  |  |
        | --- | --- |
        | Parameters: | **index** (*int*) – the index of the Force to remove |

    `getDefaultPeriodicBoxVectors`(*self*)¶
    :   Get the default values of the vectors defining the axes of the periodic box (measured in nm). Any newly created Context will have its box vectors set to these. They will affect any Force added to the System that uses periodic boundary conditions.

        |  |  |
        | --- | --- |
        | Returns: | - **a** (*Vec3*) –   the vector defining the first edge of the periodic box - **b** (*Vec3*) –   the vector defining the second edge of the periodic box - **c** (*Vec3*) –   the vector defining the third edge of the periodic box |

    `setDefaultPeriodicBoxVectors`(*self*, *a*, *b*, *c*)¶
    :   Set the default values of the vectors defining the axes of the periodic box (measured in nm). Any newly created Context will have its box vectors set to these. They will affect any Force added to the System that uses periodic boundary conditions.

        Triclinic boxes are supported, but the vectors must satisfy certain requirements. In particular, a must point in the x direction, b must point “mostly” in the y direction, and c must point “mostly” in the z direction. See the documentation for details.

        |  |  |
        | --- | --- |
        | Parameters: | - **a** (*Vec3*) – the vector defining the first edge of the periodic box - **b** (*Vec3*) – the vector defining the second edge of the periodic box - **c** (*Vec3*) – the vector defining the third edge of the periodic box |

    `usesPeriodicBoundaryConditions`(*self*) → bool¶
    :   Returns whether or not any forces in this System use periodic boundaries.

        If a force in this System does not implement usesPeriodicBoundaryConditions a OpenMM::OpenMMException is thrown

        |  |  |
        | --- | --- |
        | Returns: | true if at least one force uses PBC and false otherwise |
        | Return type: | bool |

    `getForces`()¶
    :   Get the list of Forces in this System

    `__copy__`(*self*) → System¶

    `__delattr__`¶
    :   x.\_\_delattr\_\_(‘name’) <==> del x.name

    `__format__`()¶
    :   default object formatter

    `__getattribute__`¶
    :   x.\_\_getattribute\_\_(‘name’) <==> x.name

    `__hash__`¶

    `__reduce__`()¶
    :   helper for pickle

    `__reduce_ex__`()¶
    :   helper for pickle

    `__sizeof__`() → int¶
    :   size of object in memory, in bytes

    `__str__`¶

High performance molecular simulation on GPUs


### Navigation

- Application Layer
- Library Layer
  - Core Objects
  - Forces
  - Integrators
  - Extras

©2015, Stanford University and the Authors.
|
Powered by Sphinx 1.3.1
& Alabaster 0.7.7
|
Page source
